# Deep Learning for Predicting Complex Traits in Spring Wheat Breeding Program

**DOI:** 10.3389/fpls.2020.613325

**Published:** 2021-01-05

**Authors:** Karansher S. Sandhu, Dennis N. Lozada, Zhiwu Zhang, Michael O. Pumphrey, Arron H. Carter

**Affiliations:** ^1^Department of Crop and Soil Sciences, Washington State University, Pullman, WA, United States; ^2^Department of Plant and Environmental Sciences, New Mexico State University, Las Cruces, NM, United States

**Keywords:** artificial intelligence, convolutional neural network, deep learning, genomic selection, multilayer perceptron, neural networks, wheat breeding

## Abstract

Genomic selection (GS) is transforming the field of plant breeding and implementing models that improve prediction accuracy for complex traits is needed. Analytical methods for complex datasets traditionally used in other disciplines represent an opportunity for improving prediction accuracy in GS. Deep learning (DL) is a branch of machine learning (ML) which focuses on densely connected networks using artificial neural networks for training the models. The objective of this research was to evaluate the potential of DL models in the Washington State University spring wheat breeding program. We compared the performance of two DL algorithms, namely multilayer perceptron (MLP) and convolutional neural network (CNN), with ridge regression best linear unbiased predictor (rrBLUP), a commonly used GS model. The dataset consisted of 650 recombinant inbred lines (RILs) from a spring wheat nested association mapping (NAM) population planted from 2014–2016 growing seasons. We predicted five different quantitative traits with varying genetic architecture using cross-validations (CVs), independent validations, and different sets of SNP markers. Hyperparameters were optimized for DL models by lowering the root mean square in the training set, avoiding model overfitting using dropout and regularization. DL models gave 0 to 5% higher prediction accuracy than rrBLUP model under both cross and independent validations for all five traits used in this study. Furthermore, MLP produces 5% higher prediction accuracy than CNN for grain yield and grain protein content. Altogether, DL approaches obtained better prediction accuracy for each trait, and should be incorporated into a plant breeder’s toolkit for use in large scale breeding programs.

## Introduction

Genomic selection (GS) was first proposed in animal breeding for predicting breeding values of untested individuals (Meuwissen et al., 2001). Recently, this technology has been adopted by plant breeders for predicting genomic estimated breeding values (GEBV) using genome-wide markers in GS models (Lorenzana and Bernardo, 2009; Heffner et al., 2010). GS aids in the selection of parents for use in crossing and in the selection of progenies at an earlier stage, ultimately reducing the time required for completing the breeding cycle (Jonas and De Koning, 2013; Poland, 2015). It offers the potential of increasing the genetic gain per unit time and cost by increasing selection accuracy and shortening the generation of the breeding cycle. GS has been applied in several crop species, such as barley (*Hordeum vulgare* L.), cassava (*Manihot esculenta*), maize (*Zea mays* L.), wheat (*Triticum aestivum* L.), and rice (*Oryza sativa* L.) (Maenhout et al., 2007; Isidro et al., 2015; Sallam et al., 2015; Okeke et al., 2017; Lozada and Carter, 2019). The fast-growing popularity of GS since the last decade can be attributed to the reduction in genotyping costs, producing thousands of polymorphic markers for most cultivated species (Poland et al., 2012; Wang et al., 2014). This nonetheless has resulted in a problem of so-called “large *p*, small n” when predicting phenotypes using markers.

Several statistical models are used to address this “large *p*, small n” issue by using penalized regression approaches. The most common GS model, ridge regression best linear unbiased predictor (rrBLUP), assumes markers to be random and have common variance and reduces the effect of all markers equally towards zero (Endelman, 2011). Least absolute shrinkage selection operator (LASSO) performs variable selection and continuous shrinkage simultaneously, where some markers are assumed to have an effect while others are set equal to zero (Tishbirani, 1996). Elastic net (EN) is the combination of both rrBLUP and LASSO, which uses average weight penalties from these two models (Zou and Hastie, 2005). Various Bayesian models (Bayes A, Bayes B, Bayes C, Bayes Cpi, and Bayes D) are equally important as they assume a heavy-tailed prior distribution or uses a combination of distributions for marker effects (Pérez et al., 2010; Perez-Rodriguez et al., 2012; Perez and de Los Campos, 2014). These models rely on the use of Markov Chain Monte Carlo (MCMC) for estimating the marker effects and are computationally intensive. Recently, compressed BLUP (cBLUP) and super BLUP (sBLUP) models have been developed which combines the variable selection operator of Bayes models with the computational advantage of mixed models (Wang et al., 2018). All these models are parametric as they assume a relationship between predictors and traits of interest, thus only obtaining the additive variance components, completely ignoring gene-by-gene and higher-order interactions.

Machine learning (ML) is an alternative approach for prediction and classification. ML is a branch of computer science that combines statistic and mathematic techniques for progressively training the models without explicitly programming them. ML builds different algorithms which gradually learn from the sample data and training the model, which ultimately provides predictions (Samuel, 2000). Several studies using non-parametric techniques of ML have been conducted in plants and livestock using support vector machines (SVM), boosting, random forests, and Reproducing Kernel Hilbert Space (RKHS; González-Camacho et al., 2012; González-Recio et al., 2014; Howard et al., 2014). The main advantage of using ML models for GS is that they learn the pattern from the data without being told any prior assumption, in this way they include all the variances, their interactions, and environmental components (Gianola et al., 2006; Campos et al., 2018; Gonzalez-Camacho et al., 2018). Although various studies are using ML for GS, to date, the field of deep learning (DL) has not been widely explored.

Deep learning is a branch of ML focusing on densely connected networks using artificial neural networks for training models (Min et al., 2017). The concept of DL is based on the biological networks of the brain neurons. DL uses a different combination of layers where data is transformed across each layer for obtaining a better fit. Furthermore, DL uses nonlinear activation functions, allowing them to predict the genetic architecture of the trait accurately (Angermueller et al., 2016; Wang et al., 2020). The most prominent advantage of DL is the number of high capacity and flexible trainable parameters. Traditional Bayesian neural networks are not as deep as they do not perform multiple layers of nonlinear transformation to the data (Lecun et al., 2015). DL models are continually being applied for classification and prediction problems (Pérez-Enciso and Zingaretti, 2019; Ramcharan et al., 2019). The performances of the DL algorithm have proved to be higher or similar to that of traditional ML approaches in many fields like image processing, military target recognition, genomics, speed recognition, health care, reconstructing brain circuits, traffic signal classification, and sentiment analysis (Angermueller et al., 2016; Campos et al., 2018; Bresilla et al., 2019; Zou et al., 2019). Also, there are various successful applications of DL for biological sciences, the majority of which are involved in disease classification (Rangarajan et al., 2018; Abdulridha et al., 2020).

Deep learning employs multiple neurons with proposed models such as a convolutional neural network (CNN), recurrent neural networks (RNN), and multilayer perceptron (MLP), and has the potential for application in GS (Alkhudaydi et al., 2019; Crossa et al., 2019; Cuevas et al., 2019). The input layer for these models includes a marker information, whereas the output layer consists of responses, with different number of hidden layers. Implementation of DL algorithms is straightforward, but the optimum model performance depends upon the choice of hyperparameter selection, which is not trivial and computationally intensive (Lecun et al., 2015; Young et al., 2015). Selection of hyperparameters is the most critical step for MLP, as it depends upon its ability to learn from the training data and can be generalized to a new dataset when applied for predictions. The choice of making a right decision of the number of layers, number of epochs, number of neurons, type of activation function, type of regularization penalty, activation rate, stopping criteria, among others, is cumbersome (Pérez-Enciso and Zingaretti, 2019). Optimal selection of these parameters depends upon the expertise in modeling and defining the problem. Often, the selection of parameters from a large number of tuning parameters is difficult because of time constraints and nonlinear interaction between the various parameters (Lecun et al., 2015; Young et al., 2015). There are four commonly used approaches for tuning parameters optimization, namely, random search, grid search, optimization, and Latin hypercube sampling (Koch et al., 2017). The detailed explanations of these approaches are out of the scope of this paper and are referred to in other readings (McKay, 1992; Koch et al., 2017; Montesinos-López et al., 2018a).

Several studies have focused on the use of DL models in wheat. Ma et al. (2018) have reported that CNN performs better for predicting grain length in wheat compared to traditional genomic best linear unbiased predictor (GBLUP). Similarly, Montesinos-López et al. (2018b) observed that DL models were better than GBLUP when genotype-by-environment interactions were ignored in predicting grain yield in maize and wheat. Mcdowell (2016) reported that DL models perform similarly to several linear regression and Bayesian techniques employed for GS. Although previous studies have not demonstrated a consistent advantage of DL over conventional penalized regression approaches, more efforts are required to explore the potential and constraints of DL for GS scenarios (Bellot et al., 2018; Li et al., 2018; Montesinos-López et al., 2019a; Abdollahi-Arpanahi et al., 2020). It would, therefore, be necessary to assess different DL models in the context of GS in plant breeding programs. In this study, we evaluated the performance of two different DL algorithms, namely MLP and CNN, for predicting yield, yield components, and agronomic traits having a different genetic architecture. The objectives of this study are to (1) optimize DL models for predicting complex traits in spring wheat; (2) compare the accuracy of GS for DL models with rrBLUP, one of the most commonly used GS models in plant breeding; and (3) evaluate the effect of marker number on the accuracy of the models. This study will allow us to explore the potential of DL for predicting quantitative traits in breeding programs.

## Materials and Methods

### Plant Material and Field Data

The spring wheat dataset used in this study consists of a nested association mapping (NAM) population containing 32 founder parents each crossed to common cultivar “Berkut” (Jordan et al., 2018; Blake et al., 2019). Due to space constraint, 650 Recombinant inbred lines (RILs) from 26 NAM families which have genotyping data provided by Kansas State University were planted between the 2014 and 2016 growing seasons at the Spillman Agronomy Farm near Pullman, WA, United States. A modified augmented field design was used in each trial with three replicated check cultivars [“Berkut,” “McNeal” (Lanning et al., 1994), and “Thatcher”] in each block. Five agronomic traits with varying heritability and genetic architecture, including grain yield, grain protein content, heading date, plant height, and test weight were evaluated. Grain yield (t/ha) was calculated using a Wintersteiger Nursery Master combine (Ried im Innkreis, Austria) from grain weight per plot by harvesting whole plots. A Perten DA 7000 NIR analyzer (Perkin Elmer, Sweden) was used to determine the percentage of protein content in the grain. Days to heading was recorded as the number of days from planting to full exposure of spikes in 50% of the plot. Plant height (cm) was measured as length between the base of the plant to the tip of the fully emerged spike, excluding the awn when present. Test weight (kg hL^−1^) was measured postharvest (Perkin Elmer, Sweden).

### Statistical Analysis

Adjusted means were calculated for the unreplicated genotypes using the residuals derived separately for the individual environment using “lme4” function implemented in the R program using the model:

$$Y_{\mathrm{ij}}={\text{Block}}_{i}+{\text{Check}}_{j}+{\text{residuals}}_{\mathrm{ij}}$$

where *Y_ij_* is the trait of interest, Block*_i_* is the fixed effect of the *i*th block, and Check*_j_* corresponds to the effect of replicated check cultivar (Bates et al., 2015; R Core Team, 2017).

Broad-sense heritability for all phenotypic data points were calculated for each environment separately using the formula:

$$H^{2}=\sigma^{2}{}_{\text{g}}/\left(\sigma^{2}{}_{\text{g}}+\sigma^{2}{}_{\text{e}}\right)$$

where *H*^2^ is the broad-sense heritability, *σ*^2^*_g_* and *σ*^2^*_e_* are the genotypic and error variance components, respectively, obtained from the augmented randomized complete block design model treating genotype effects as random using the model equation:

$$Y_{\mathrm{ij}}=\mu +{\text{Block}}_{i}+{\text{Check}}_{j}+{\text{Gen}}_{\text{j}\left(\text{i}\right)}+e_{\mathrm{ij}}$$

where *Y_ij_* is the trait of interest, Block*_i_* is the fixed effect of the *i*th block, Gen*_j_* is the random effect of unreplicated genotypes *j* nested within *i*th block and distributed as independent and identically distributed, Gen*_j_* ~ *N*(0, *σ*^2^*_g_*), Check*_j_* corresponds to effect of replicated check cultivar, and *e_ij_* is the standard normal errors distributed as *e_ij_* ~ *N*(0, *σ*^2^*_e_*) (Federer, 1961; Aravind et al., 2020).

### Genotyping

The NAM population was genotyped using the Illumina 90 K SNP array (Wang et al., 2014) and genotyping-by-sequencing (GBS; Poland et al., 2012). Information on genotyping, map construction, and marker calling has been previously reported (Jordan et al., 2018). The initial genotypic information consisted of 73,345 polymorphic markers anchored to the Chinese Spring RefSeqv1 map (International Wheat Genome Sequencing Consortium, 2014; Jordan et al., 2018). RILs with missing phenotypic information in one environment were removed before filtering the genotypic data. SNP markers with more than 20% missing data, minor allele frequency of <0.10, and RIL missing >10% genotypic data were also discarded, resulting in a total of 635 RILs with 40,000 SNP markers used for analyses. Principal component analysis (PCA) was performed for assessing the population structure among the 26 NAM families using 40,000 SNP markers and 635 RILs. The whole data set and filtering pipeline used is provided on GitHub.^1^

### Genomic Selection Models

#### Penalized Regression Models

Ridge regression best linear unbiased predictor is one of the most used GS models in plant breeding and was included here for comparison with the DL algorithms. Genome-wide marker effects were estimated using rrBLUP model for all traits (Endelman, 2011). GEBVs were calculated with mixed solve function implemented in R package “rrBLUP,” according to the model:

y=μ+Zu+e

where *y* is an *N* × 1 vector of adjusted means for all unreplicated genotypes, *μ* is the overall mean, *Z* is an *N* × *M* matrix assigning markers to genotypes, *u* is a vector with normally distributed random marker effects as *u* ~ *N*(0, *I𝝈*^2^*_u_*), and *e* is the residual error with *e* ~ *N*(0, *I𝝈*^2^*_e_*). The solution for mixed equation can be written as

$$u=Z^{T}{\left(ZZ^{T}+\lambda I\right)}^{-1}y$$

where *λ* is the ridge regression parameter represented as *λ* = *𝝈*^2^*_e_*/*𝝈*^2^*_u_* is the ratio of residual and marker variances. rrBLUP has the potential for dealing with “large *p* and small n” with penalized regression and has high numerical stability with highly correlated markers (Hoerl and Kennard, 2000). Codes and data set used for implementing the rrBLUP GS model is uploaded at GitHub.^1^

#### Multilayer Perceptron

**Figure 1:**
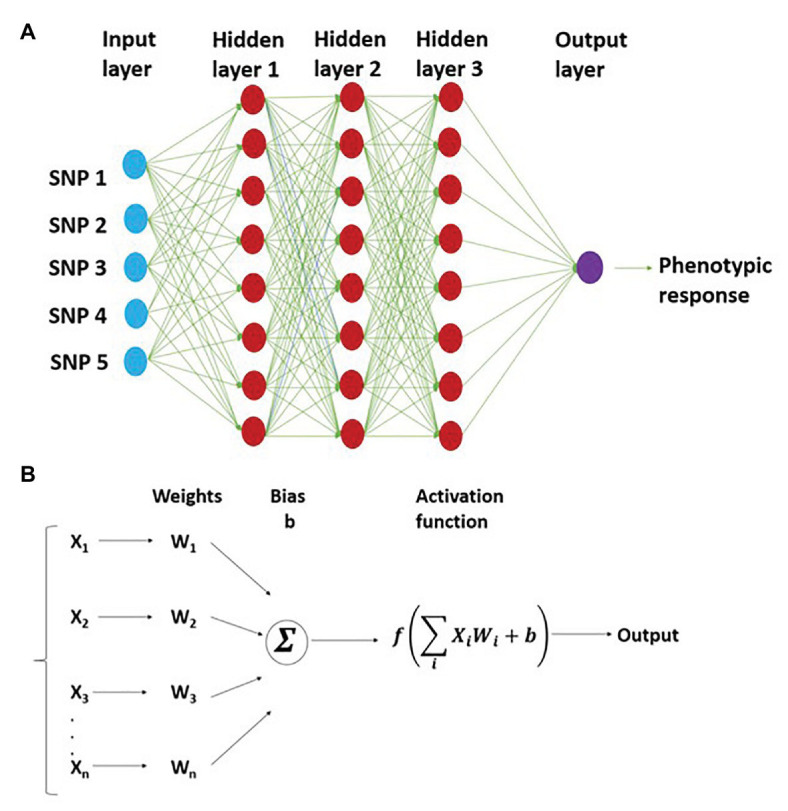
Representation of multilayer perceptron (MLP) with three hidden layers and five SNP markers in the input layer. This shows the network structure for working of MLP, where the connection between different neurons is depicted **(A)**; bottom half represents weight assigned to each neuron and prediction of output using nonlinear activation function **(B)**.

Multilayer perceptron is a densely connected network, which is a typical feedforward neural network and does not assume a particular structure in the input features (Gulli and Pal, 2017). The basic structure of MLP consists of a densely connected network of the input layer, output layer, and multiple hidden layers (Figure 1). All these layers are connected by a dense network of neurons, where each neuron has its characteristic weight (Angermueller et al., 2016). In the case of GS, the input layer consists of a certain fixed number of neurons where each neuron represents an SNP marker in the training set. There are multiple hidden layers with a different number of neurons. Different layers are connected by neurons with a strength called “weight.” The weight coefficient of neurons between the input and output layers is obtained from the training dataset using non-linear transformations. The number of output layer neurons is equal to the number of response variables in the GS model.

During the GS model training, the output of hidden layer one is a weighted average nonlinear transformation function of each input plus a bias (*b*; Figure 1). The output of the first layer (hidden layer 1) is represented as

$$Z_{1}=b_{0}+W_{0}f_{0}\left(x\right)$$

where *Z*_1_ is the output of the first layer, *b*_0_ is the bias for the first layer estimated from the rest of the weights (*W*_0_)_,_ *x* represents the genotypes of each individual, and *f* is a nonlinear activation function. This model is trained successively, where the output of neurons from the previous layer act as input for the next layer. The general expression for the model is

$$Z_{\text{k}}=b_{\text{k}-1}+W_{\text{k}-1}f_{\text{k}-1}\left(x\right)$$

where *Z_k_* is the output vector for the GEBVs, and other terms of this equation are defined previously.

#### Convolutional Neural Network

**Figure 2:**
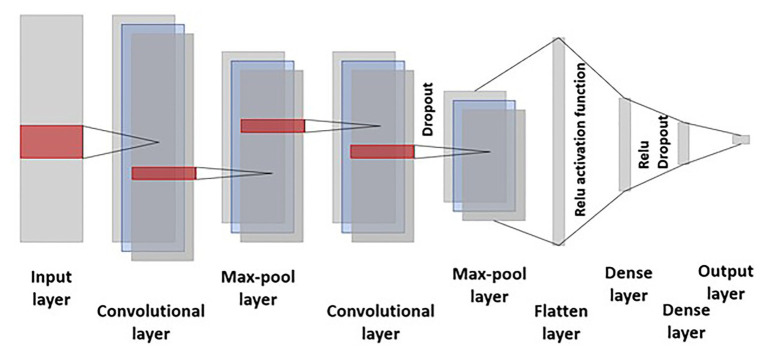
Representation of the convolutional neural network (CNN) employed in this study. The input layer consists of 40,000 markers with a kernel size of three in the convolutional layer. Dropout is employed after the second convolutional and first dense layer. Relu activation function was used for training the model and hyperparameters were selected by lowering the mean squared error.

Convolutional neural network is proposed to accommodate inputs that are associated with each other such as linkage disequilibrium between nearby SNP markers. A CNN is a special case of artificial neural networks where hidden layers typically consist of convolutional layers, pooling layers, flatten layers, and fully connected dense layers. In each convolutional layer, CNN automatically performs the convolution operation along with an input of predefined width and strides through the application of kernels and filters where the weights are the same for all SNP marker windows. The filter moves for the same window size across the input SNP markers, and CNN obtains the local weighted sum. The learned filters move across the input SNP marker data until the entire genotypic data are transverse. Each of these convolutional operations learns the coefficient of the so-called “kernel” or filter, which is equivalent to neurons of MLP. The output of the convolutional function can be defined as an integral transformation and is represented as

$$s\left(t\right)=\left(f*k\right)\left(t\right)=\sum_{x}k\left(t-x\right)f\left(x\right)$$

where *k* represents the kernel, convolution is the transformation of *f* into *s(t)*, and this operation is performed over an infinite number of copies *f* shifting over the kernel along each chromosome and filters take into account the linkage disequilibrium along the chromosome. A max-pooling layer is added after each convolutional layer to account for dimensionality reduction and making filters invariant to the small changes in the input. The pooling layer smoothed out the results by merging the output of the previous convolutional layer by taking the minimum, mean, and maximum. Activation function and dropout is employed after the convolutional and dense layer (Figure 2).

The greatest advantage of CNN over MLP is their capability to reduce the estimation of the number of hyperparameters required for training the model. Successive output layers are produced by the action of the activation function over the previous convolution layer. Finally, the pooling operation is performed resulting in dimension reduction, smoother representation, and merging of kernel output by computing their mean, maximum, or minimum.

#### Hyperparameter Optimization

A grid search cross-validation (CV), which selects the parameters that provide minimum mean square error (MSE; Pedregosa et al., 2011; Cho and Hegde, 2019) was implemented to optimize the hyperparameters on the whole population and for all traits evaluated in this study. Based upon available literature, we selected hyperparameters for training, and based on those parameters, a grid search CV with the full factorial design was implemented. The different hyperparameters which were tried for optimizing includes learning rate (constant and adaptive), activation function (relu, linear, tanh, identity, and logistic), solver (lbfgs, sgd, and adam), number of hidden layers (1, 4, 6, 8, and 10), number of neurons in completely dense network (10, 19, 38, 50, 62, 98, 112, and 150), drop out (0, 0.01, 0.1, and 0.2), number of filters (16, 32, 64, and 128), and regularizations (L1 and L2). Grid search CV used the inner CV where the outer training data set was split to 80% for inner training and the remaining 20% for inner testing. The inner training data set was used for hyperparameter optimization using the Keras validation split function and internal capabilities. The best hyperparameters were selected that give the least MSE on the inner testing population, and hence those parameters were used for the individual traits (Gulli and Pal, 2017).

Overfitting, which is related to poor model performance on the validated set, is one of the biggest constraints in implementing DL strategies in plant breeding. With this, approaches such as regularization, dropout, and early stopping were applied to minimize overfitting in the models. Dropout includes randomly assigning a subset of training neuron’s weight to zero to reduce complexity and overfitting. Herein, we used a 0.2 fixed dropout rate during hyperparameter optimization based on Srivastava et al. (2014) and Early stopping involves terminating the training process depending on the validation performance. As soon as the validation error reaches a minimum, training is halted. Keras provides an API (Callbacks) to incorporate the feature of early stopping. We used the EarlyStopping callback to create our MLP and CNN model. The other regularization techniques, L1 and L2, penalize weight values of the neural network. This technique involves making values close to zero and negative equal to 0 as they do not affect the model’s performance. L1 penalizes the sum of the absolute values of weights, whereas L2 penalizes the weight’s sum of the square. Our analysis made use of the parameter alpha of MLP and added L1_L2 regularizer in the first convolutional layer of CNN model. The DL algorithms were implemented in Scikit learn and Keras in Spyder (Python 3.7; Pilgrim and Willison, 2009; Pedregosa et al., 2011; Gulli and Pal, 2017). Codes and data set used for implementing the DL models is uploaded at GitHub.^1^

#### Cross-Validation and Independent Prediction

Prediction accuracy for the GS models (rrBLUP, MLP, and CNN) was evaluated by implementing a five-fold CV where 80% of the data was included in the training population, and 20% of the remaining data was used as a testing set within each environment. Two hundred replications were performed for each model to assess model performance. Each replication consisted of five iterations, where the dataset was split into five groups, and a different testing set was used for each iteration. Instant accuracy was calculated where correlation for each testing set was obtained and an average of five iterations was reported. Accuracy of the GS model was defined as the Pearson correlation coefficient between GEBVs and true (observed) phenotypes. A total of nine random sets of markers were used for training models and comparing the effect of marker number on the model’s performance, including 1,000 (M_1,000_), 5,000 (M_5,000_), 10,000 (M_10,000_), 15,000 (M_15,000_), 20,000 (M_20,000_), 25,000 (M_25,000_), 30,000 (M_30,000_), 35,000 (M_35,000_), and 40,000 (M_40,000_) SNP markers, and these models were also implemented using 200 replications with five-fold CV.

Independent validation was performed by training the GS model on the previous growing season, and predictions were made for future years. Briefly, the GS model was trained on the 2014 environment, and the prediction was made for the 2015 and 2016 environments. Similarly, the GS model trained on 2015 environment was used for predicting the 2016 environment. This type of validation represents the scenario of predicting the performance of a line before planting them in the field for the next growing season. Due to the computational burden of DL models, the whole analysis was completed on the WSU’s high computing cluster.^2^ When implemented on a single system, MLP and CNN were 40- and 55-fold more time-consuming. We solved this issue by executing the iterations in parallel on the cluster computers.

## Results

### Heritability and Population Structure

Broad-sense heritability for all the five traits was obtained for each environment (Table 1). Each trait had different heritability values, depicting different genetic makeup, and varying environmental effects. Plant height and heading date were highly heritable, grain protein content, and test weight were moderately heritable, and grain yield was the least heritable among the traits. The heritability of each trait was lowest for the 2015 environment suggesting a more non-genetic variance effect for that environment. PCA showed the presence of two subgroups in population where PC1 and PC2 explained 5 and 4% of total genetic variation, respectively (Supplementary Figure 1). Furthermore, PC1 and PC2 for five different phenotypic traits evaluated in this study explained 31.8 and 21.4% of the variation (Supplementary Figure 2). In PC1, grain protein content and days to heading were clustered together and were opposite from test weight and grain yield.

**Table 1:** Broad-sense heritability of five different traits for each environment (2014–2016) evaluated in this study.

Environment	Grain yield	Grain protein content	Test weight	Plant height	Heading date
2014	0.38	0.57	0.68	0.81	0.84
2015	0.24	0.35	0.59	0.59	0.80
2016	0.40	0.63	0.57	0.89	0.91

### Optimization of Hyperparameters for Each Trait

Different hyperparameters for each trait were selected using a grid search CV for 200 iterations by lowering the MSE. The combinations of hyperparameters were selected for each trait that had the lowest MSE during 200 iterations of grid search CV. These selected hyperparameters were used for predicting the traits for each environment separately. All the hyperparameters chosen in this study are provided for MLP (Table 2) and CNN (Table 3). The number of filters was the most important factor for lowering MSE in the case of CNN. In the case of MLP, activation function and number of neurons in layers were the main parameters controlling model performance. Different dropout and regularization values were selected to reduce overfitting in the model by looking at training accuracy, and these values were used for the testing set (Tables 2 and 3). We provided the information about the hyperparameters required for tuning each trait separately because of the different genetic architecture of the five traits used in this study. These results were consistent with other studies which also showed that different hyperparameters are required for various traits in plant breeding (Cuevas et al., 2019; Montesinos-López et al., 2019b).

**Table 2:** Hyperparameters selected for each trait using a random grid search CV for MLP.

Hyperparameter	Grain yield	Grain protein content	Test weight	Plant height	Heading date
Activation function	relu	relu	relu	tanh	tanh
Solver	adam	adam	sgd	sgd	sgd
Learning rate	Adaptive	Adaptive	Constant	Constant	Constant
No. of hidden layers	4	4	4	3	3
No. of neurons	(38, 38, 38, 19)	(19, 19, 19)	(50, 38, 38)	(120, 90, 90)	(90, 90, 90)
Dropout	0.2	0.2	0.2	0.2	0.2
Epochs	200	200	200	150	150
Regularization	0.1	0.1	0.05	0.05	0.05

These hyperparameters were later used for training the models.

**Table 3:** Hyperparameters selected for each trait using a random grid search CV for CNN.

Hyperparameter	Grain yield	Grain protein content	Test weight	Plant height	Heading date
Activation function	relu	relu	relu	relu	relu
Solver	adam	adam	adam	adam	adam
Learning rate	adaptive	adaptive	constant	constant	constant
Number of filters	64	64	64	64	64
Dropout	0.2	0.2	0.2	0.2	0.2
Epochs	200	200	200	200	200
Regularization	0.05	0.05	0.05	0.05	0.05

These hyperparameters were later used for training the models.

### Comparison of Model Performances for Cross-Validations

**Figure 3:**
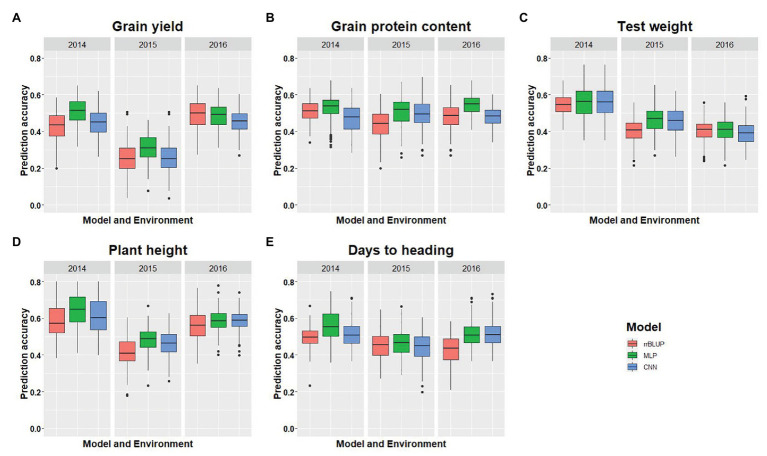
Comparison of model performance for five different traits used in this study. **(A-E)** represent the model’s performance for grain yield, grain protein content, test weight, plant height, and days to heading, for each trait under each environment using five-fold cross-validation (CV) and 40,000 SNP markers. The *x*-axis represents the environment, and the *y*-axis represents the prediction accuracy for the model.

We compared the performances of two DL models with rrBLUP for each of the environments using the whole marker dataset (M_40,000_) for GS. Figure 3 shows the prediction accuracy for each of the five traits with three models, namely rrBLUP, MLP, and CNN under each environment. Furthermore, average prediction accuracy over the environment for each model is provided for all five traits (Table 4). Average prediction accuracy was highest with MLP for all the five traits. MLP improves the prediction accuracy from 3 to 5% for all the traits compared to rrBLUP, which is the most often used model in wheat breeding for predicting quantitative traits (Rutkoski et al., 2011; Sun et al., 2019). Even CNN gave 0 to 3% higher prediction accuracy than rrBLUP (Table 4). These results suggest that DL models should be included to obtain slightly higher prediction accuracies, as even minor increases in prediction accuracy could improve the selection efficiency in a breeding program. The improvement in prediction accuracy with DL models compared to the linear rrBLUP model is attributed to the use of nonlinear activation functions relu and tanh, which model the nonlinear relationship and ignore the restrictive assumptions of rrBLUP.

**Table 4:** Comparison of average prediction accuracy with three models (rrBLUP, MLP, and CNN) for five traits evaluated in this study and predicted separately for each environment for spring wheat.

Model	Grain yield	Grain protein content	Test weight	Plant height	Heading date
rrBLUP	0.39	0.48	0.45	0.52	0.46
MLP	**0.44**	**0.53**	**0.48**	**0.57**	**0.51**
CNN	0.39	0.48	0.47	0.55	0.49

The highest prediction accuracy is bolded for each trait under each model scenario.

Multilayer perceptron gave 5% higher prediction accuracy than CNN for grain protein content and grain yield (Table 4). Among the five tested traits, grain yield and grain protein content are controlled by a large number of QTL, and high prediction accuracy with MLP is due to use of more hidden layers and less number of neurons which more efficiently capture the complex relationship between the SNP markers and response (Table 2; Sukumaran et al., 2015; Arora et al., 2017). Furthermore, both MLP and CNN performed similarly for predicting test weight, plant height, and days to heading. This suggests that either of these models could be used for predicting those traits in spring wheat. Furthermore, some hyperparameters are specific for particular traits (Tables 2 and 3). Grain yield and grain protein content requires a greater number of hidden layers compared to the other three traits, demonstrating that complex DL networks are required for highly quantitative traits (Bellot et al., 2018).

Complete details about prediction accuracy for each model on each environment is provided in full detail in Figure 3. There was a difference in prediction accuracy for each trait with all the models under different environmental conditions. This is because of the different heritability of each trait across the environments and the varying amounts of genetic variances captured by each model. Furthermore, DL models were able to capture the different amount of environmental variance as shown in Figure 3A, where the rrBLUP and DL models performed similarly for the 2016 environment, whereas for 2014, MLP had an 8% higher prediction accuracy than rrBLUP, suggesting that more environmental effect was captured. Similar trends can be explained for all the other traits predicted in this study (Figure 3).

### Marker Set Optimization

**Figure 4:**
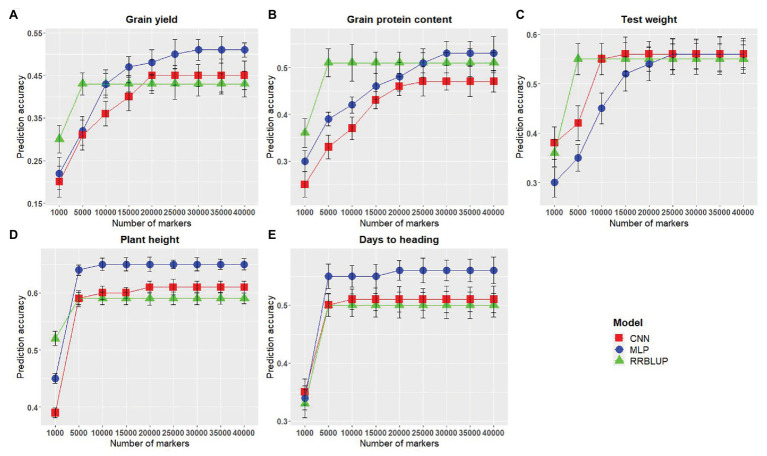
Comparison of markers numbers for each of the genomic selection (GS) models for predicting five different traits in wheat. **(A-E)** represent the model performances for grain yield, grain protein content, test weight, plant height, and days to heading, respectively. The *x*-axis represents the number of markers in the model, and the *y*-axis represents prediction accuracy.

The number of predictors (markers) has been reported to have a significant effect on the GS model performance (Heffner et al., 2011; Ma et al., 2018; Lozada and Carter, 2019). Therefore, we assessed the effect of the number of SNP markers on the performance of GS models evaluated in this study. Across all models, an increased marker number was related to improved prediction accuracy. The lowest prediction accuracy was obtained using M_1,000_ for all the evaluated traits (Figure 4). Non-significant differences in model performances were observed when the marker number was increased from M_5,000_ to M_40,000_ for rrBLUP (Figure 4). MLP and CNN models rendered consistent improvement in prediction accuracy as the number of markers were increased in the model (Figure 4); nevertheless, trends vary across traits. Accuracy for plant height and days to heading reached a stable value when M_5,000_ or more markers were used for MLP and M_10,000_ or more markers were included in CNN model (Figures 4D,E). This can be attributed to a small number of QTLs which are controlling these traits; hence, this number of markers is able to capture all of them efficiently. In the case of test weight, there was a consistent increase in prediction accuracy for MLP and CNN until marker numbers reached above M_15,000_, where no further significant increase in accuracy was observed (Figure 4C). Prediction accuracy for grain protein content and grain yield continuously increased as markers were increased to M_30,000_ for MLP and M_25,000_ for CNN (Figures 4A,B). These results suggest that with the reduction in genotyping cost, which produces a plethora of genotyping information, DL models should be used to obtain an increased prediction accuracy by efficiently using a large number of predictors in the GS models.

### Prediction Accuracy Across Environments

**Figure 5:**
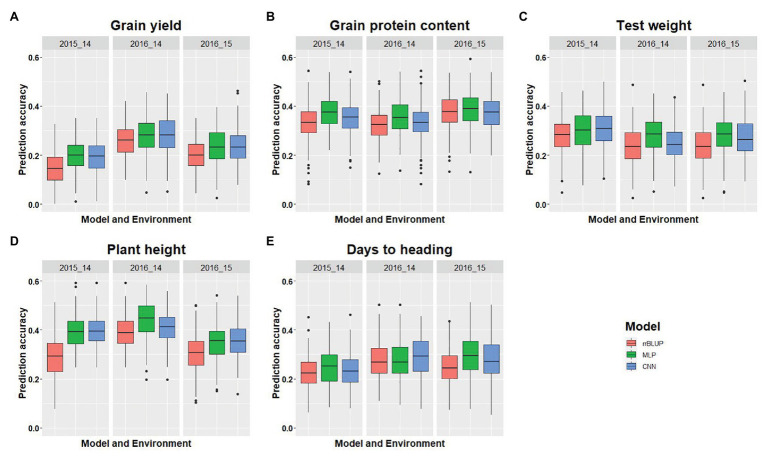
GS model performance for independent validation using all three models for predicting five different traits in wheat **(A-E)**. The *x*-axis represents the environments where predictions were implemented. The first year indicates the testing, whereas the second year is the training environment.

In addition to looking at prediction accuracy within environments, model performance in an across-environment prediction scenario was also assessed. GS models were trained on data from the previous year, and predictions were made for next year phenotypic data. Average prediction accuracy for the independent validations for all five traits is provided (Table 5). Figure 5 shows the prediction accuracy for each of the five traits with three tested models under each environmental condition when the model was trained on the previous year dataset. There was a significant decrease in prediction accuracy under independent validation compared to CV for each trait (Tables 4 and 5). This is because of using different populations for training and testing the model, which results in a different amount of non-genetic variances. Independent validations could be potentially improved by inclusion of genotype by environmental interactions in the model or by inclusion of more phenotypic data in the training models (Heffner et al., 2010; Lorenz et al., 2011). Furthermore, DL models performed equal or slightly better than rrBLUP for all the traits and strengthens the findings from the CV analysis (Table 5).

**Table 5:** Comparison of average prediction accuracy under the independent validation scenario with three models (rrBLUP, MLP, and CNN) for five traits evaluated in this study for spring wheat.

Model	Grain yield	Grain protein content	Test weight	Plant height	Heading date
rrBLUP	0.20	0.34	0.25	0.33	0.25
MLP	**0.24**	**0.37**	**0.29**	**0.39**	**0.27**
CNN	0.23	0.35	0.28	**0.39**	**0.27**

The highest prediction accuracy is bolded for each trait under each model scenario.

## Discussion

Genomic selection is transforming the field of plant breeding, and therefore using models with increased predictive power is relevant. DL is a new ML-based technique which explores the complex relationships hidden in the data for making predictions. In this study, we investigated the application of DL-based GS models for predicting complex traits in spring wheat. DL approaches were successfully applied for predictions and optimization of hyperparameters for each trait. Higher prediction accuracy (0–5%) with DL models compared to rrBLUP were observed for predicting five traits. Using a different number of markers in the model influenced the accuracy of GS for the evaluated traits, where an improved accuracy was related to increased marker number.

The optimization of hyperparameters for DL models is critical and challenging because of the high computational costs in this study, nevertheless, this optimization issue was solved using grid search CV (Young et al., 2015). First, we observed that each trait requires various combinations of hyperparameters, as prediction accuracy is dependent upon the interaction of these factors (Bellot et al., 2018; Montesinos-López et al., 2018b). The different tuning parameters for each trait depend on the genetic architecture of the trait. We observed that the “relu” activation function was the most important for predicting all traits in CNN and most of the traits in MLP, suggesting that “relu” function is critical for training GS models in wheat. Several studies have validated this function as a universal function for regression-based prediction models (Lecun et al., 2015; Pérez-Enciso and Zingaretti, 2019). Furthermore, different layers in CNN (convolutional, max-pooling, dense and fully connected) require a different set of hyperparameters, thus creating challenges in understanding the complex biological connection (Min et al., 2017). We obtained a higher prediction accuracy with DL, but those results are only valid for the hyperparameters used in this study (Montesinos-López et al., 2018b).

The high prediction accuracy of DL models compared to rrBLUP under both cross- and independent-validation scenarios can be attributed to the presence of hidden layers which automatically captures the complex hidden interaction without prior specification (Lecun et al., 2015). This means that unlike rrBLUP, which only models first-order interactions, DL models can capture interactions of large orders without specifying so in the model. DL could therefore explore data in such a way that humans cannot see and extract conclusions which otherwise are not possible to catch (Goodfellow et al., 2016). Higher or equal prediction accuracy of DL with rrBLUP for all the traits suggest that these models should be further explored in wheat, to further improve the prediction accuracy with inclusion of secondary correlated traits and genotype-by-environment interaction effects (Cuevas et al., 2019; Montesinos-López et al., 2019b).

It should be noted that rrBLUP was competitive with the DL models in terms of the accuracy of GS in the current study. The rrBLUP model’s interpretability, transparency, and absence of the time-consuming task of hyperparameter tuning still makes it an attractive approach for GS, though the potential of improving prediction accuracy using DL approaches could not be discounted. Ma et al. (2018) reported that DL-based methods performed better than rrBLUP for predicting grain length, grain hardness, plant height, grain protein, and thousand kernel weight in wheat. They further suggested that both DL and rrBLUP models should be used for selecting the “best” individuals. Our results were consistent with their observations that DL approaches give slightly better prediction accuracy than rrBLUP, but with some computational costs associated with the DL models. Montesinos-López et al. (2018b) on the other hand observed DL models to be superior compared to GBLUP in six out of the nine traits evaluated in wheat and maize. Liu et al. (2019) also demonstrated the superiority of single and dual CNN models over the rrBLUP for predicting yield, protein, oil, moisture, and height in soybean (*Glycine max* L.). Similarly, Zingaretti et al. (2020) showed that DL models perform better than conventional linear statistical models for predicting traits having epistatic variances in the allopolyploid species of strawberries (*Fragaria x ananassa*) and blueberries (*Cyanococcus* spp.). These and our results open the field of DL in plant breeding and suggest that there is a great potential to increase predictive power for complex traits using DL approaches.

The performance of DL models improves when a large dataset is used for training the model (Min et al., 2017). Our current results and some related works, nonetheless, support that DL based models can reach an equivalent or superior accuracy than traditional linear models for GS even with the smaller dataset for training (Ma et al., 2018; Montesinos-López et al., 2018a). Furthermore, a previous study using the largest dataset analyzed so far (100 k individuals) for training the DL model does not provide the superiority over the linear models (Bellot et al., 2018). These results altogether suggest that training population size is less important compared to the trait used for the prediction; this does not however undermine the use of large population sizes in the GS model. The biggest issue with a small dataset for DL is overfitting, which results from the failure of the model to learn general patterns present in the data. We tried to avoid overfitting in our models using dropout and regularization, which involves the removal of some fixed number of neurons during model training (Lecun et al., 2015; Bellot et al., 2018).

One drawback of DL models is that different hyperparameters handle different parts of the data, resulting in a problem for interpreting biological significance and importance of each feature (marker) in the model (Bellot et al., 2018; Cuevas et al., 2019). DL models, consequently, might not be useful for providing insights into the genetic architecture of the trait; instead, genome-wide association studies might be more appropriate for this purpose. Furthermore, the computational cost is a significant hindrance for training DL models, as multiple hyperparameters are required to be optimized for each trait separately (Gulli and Pal, 2017; Cho and Hegde, 2019). Plant scientists are often interested in understanding the biological meaning of prediction models, which is difficult in DL-based models because of the “black-box” nature of neural networks, and a large number of layers and neurons involved in training the model. Finally, DL based models require a background in computer science and statistics, which might require additional expertise or collaborations. Nevertheless, despite these limitations, DL approaches could still be used in the context of GS in plant breeding programs. Overall, this study opens a new avenue of DL for the prediction of complex traits in plant breeding.

## Conclusion

In this study, we compared the performance of two DL models, namely MLP and CNN, with rrBLUP for predicting five different traits in spring wheat. Our results suggest that DL based models are superior for predicting all five traits used in this study. We optimized the hyperparameters required for training different traits and validated that each trait requires a specific set of hyperparameters for best performance. We observed that prediction accuracy for DL models was trait dependent and improved as the number of predictors (markers) in the models increased. Although training the DL models is computationally intensive and challenging, we found that the application of DL-based approaches is feasible and promising in terms of improving the prediction accuracy for complex traits in spring wheat. For these reasons, DL models should be incorporated into a plant breeder’s toolkit for use in large scale breeding programs to improve genetic gain for quantitative traits.

## Data Availability Statement

The source codes and datasets used are made available on the GitHub account, and a link is provided in the manuscript.

## Author Contributions

KS analyzed data, conceptualized the idea, and drafted the manuscript. DL edited the manuscript. ZZ assisted in data analysis and edited the manuscript. MP and AC edited the manuscript, conducted field trials, and obtained the funding for the project. All authors contributed to the article and approved the submitted version.

### Conflict of Interest

The authors declare that the research was conducted in the absence of any commercial or financial relationships that could be construed as a potential conflict of interest.

## Footnotes

^1^https://github.com/Sandhu-WSU/DL_Wheat.git

^2^https://hpc.wsu.edu/

## References

[ref1] Abdollahi-Arpanahi R. Gianola D. Peñagaricano F. (2020). Deep learning versus parametric and ensemble methods for genomic prediction of complex phenotypes. Genet. Sel. Evol. 52:12. 10.1186/s12711-020-00531-z, PMID: 32093611 PMC7038529

[ref2] Abdulridha J. Ampatzidis Y. Roberts P. Kakarla S. C. (2020). Detecting powdery mildew disease in squash at different stages using UAV-based hyperspectral imaging and artificial intelligence. Biosyst. Eng. 197, 135–148. 10.1016/j.biosystemseng.2020.07.001

[ref3] Alkhudaydi T. Reynolds D. Zhou J. Iglesia B. Griffiths S. (2019). An exploration of deep-learning based phenotypic analysis to detect spike regions in field conditions for UK bread wheat. Plant Phenom. 2019:7368761. 10.34133/2019/7368761 PMC7706304 33313535

[ref4] Angermueller C. Pärnamaa T. Parts L. Stegle O. (2016). Deep learning for computational biology. Mol. Syst. Biol. 12:878. 10.15252/msb.20156651, PMID: 27474269 PMC4965871

[ref5] Aravind J. Mukesh S. S. Wankhede D. P. (2020). AugmentedRCBD: Analysis of augmented randomised complete block designs. R package version 0.1.3.

[ref6] Arora S. Singh N. Kaur S. Bains N. S. Uauy C. Poland J.. (2017). Genome-wide association study of grain architecture in wild wheat *Aegilops tauschii*. Front. Plant Sci. 8:886. 10.3389/fpls.2017.00886, PMID: 28620398 PMC5450224

[ref7] Bates D. Mächler M. Bolker B. Walker S. (2015). Fitting linear mixed-effects models using lme4. J. Stat. Softw. 67, 1–48. 10.18637/jss.v067.i01

[ref8] Bellot P. de los Campos G. Pérez-Enciso M. (2018). Can deep learning improve genomic prediction of complex human traits? Genetics 210, 809–819. 10.1534/genetics.118.301298, PMID: 30171033 PMC6218236

[ref9] Blake N. K. Pumphrey M. Glover K. Chao S. Jordan K. Jannick J. L. (2019). Registration of the triticeae-cap spring wheat nested association mapping population. J. Plant Regist. 13, 294–297. 10.3198/jpr2018.07.0052crmp

[ref10] Bresilla K. Perulli G. D. Boini A. Morandi B. Corelli Grappadelli L. Manfrini L. (2019). Single-shot convolution neural networks for real-time fruit detection within the tree. Front. Plant Sci. 10:611. 10.3389/fpls.2019.00611, PMID: 31178875 PMC6537632

[ref11] Cho M. Hegde C. (2019). “Reducing the search space for hyperparameter optimization using group sparsity” in *ICASSP, IEEE International Conference on Acoustics, Speech and Signal Processing - Proceedings*, May 12–17, 2019 (Institute of Electrical and Electronics Engineers Inc.), 3627–3631.

[ref12] Crossa J. Martini J. W. R. Gianola D. Pérez-rodríguez P. Jarquin D. Juliana P.. (2019). Deep kernel and deep learning for genome-based prediction of single traits in multienvironment breeding trials. Front. Genet. 10:1168. 10.3389/fgene.2019.01168, PMID: 31921277 PMC6913188

[ref13] Cuevas J. Montesinos-lópez O. Juliana P. Guzmán C. Pérez-rodríguez P. González-bucio J.. (2019). Deep kernel for genomic and near infrared predictions in multi-environment breeding trials. G3 (Bethesda) 9, 2913–2924. 10.1534/g3.119.400493, PMID: 31289023 PMC6723142

[ref14] de los Campos G. Vazquez A. I. Hsu S. Lello L. (2018). Complex-trait prediction in the era of big data. Trends Genet. 34, 746–754. 10.1016/j.tig.2018.07.004, PMID: 30139641 PMC6150788

[ref15] Endelman J. B. (2011). Ridge regression and other kernels for genomic selection with R package rrBLUP. Plant Genome 4, 250–255. 10.3835/plantgenome2011.08.0024

[ref16] Federer W. T. (1961). Augmented designs with one-way elimination of heterogeneity. Int. Biom. Soc. 17, 447–473.

[ref17] Gianola D. Fernando R. L. Stella A. (2006). Genomic-assisted prediction of genetic value with semiparametric procedures. Genetics 173, 1761–1776. 10.1534/genetics.105.049510, PMID: 16648593 PMC1526664

[ref18] González-Camacho J. M. de los Campos G. Pérez P. Gianola D. Cairns J. E. Mahuku G.. (2012). Genome-enabled prediction of genetic values using radial basis function neural networks. Theor. Appl. Genet. 125, 759–771. 10.1007/s00122-012-1868-9, PMID: 22566067 PMC3405257

[ref19] González-Camacho J. M. Ornella L. Pérez-Rodríguez P. Gianola D. Dreisigacker S. Crossa J. (2018). Applications of machine learning methods to genomic selection in breeding wheat for rust resistance. Plant Genome 11:170104. 10.3835/plantgenome2017.11.0104, PMID: 30025028 PMC12962436

[ref20] González-Recio O. Rosa G. J. M. Gianola D. (2014). Machine learning methods and predictive ability metrics for genome-wide prediction of complex traits. Livest. Sci. 166, 217–231. 10.1016/j.livsci.2014.05.036

[ref21] Goodfellow I. J. Bengio Y. Courville A. C. (2016). Deep learning. Adaptive computation and machine learning. Cambridge: MIT Press.

[ref22] Gulli A. Pal S. (2017). Deep learning with Keras. Birmingham: Packt Publishing Ltd.

[ref23] Heffner E. L. Jannink J. L. Sorrells M. E. (2011). Genomic selection accuracy using multifamily prediction models in a wheat breeding program. Plant Genome 4, 65–75. 10.3835/plantgenome2010.12.0029

[ref24] Heffner E. L. Lorenz A. J. Jannink J. L. Sorrells M. E. (2010). Plant breeding with genomic selection: gain per unit time and cost. Crop Sci. 50, 1681–1690. 10.2135/cropsci2009.11.0662

[ref25] Hoerl A. E. Kennard R. W. (2000). Ridge regression: biased problems nonorthogonal estimation for nonorthogonal problems. Technometrics 42, 80–86.

[ref26] Howard R. Carriquiry A. L. Beavis W. D. (2014). Parametric and nonparametric statistical methods for genomic selection of traits with additive and epistatic genetic architectures. G3 (Bethesda) 4, 1027–1046. 10.1534/g3.114.010298, PMID: 24727289 PMC4065247

[ref40] International Wheat Genome Sequencing Consortium (2014). A chromosome-based draft sequence of the hexaploid bread wheat (*Triticum aestivum*) genome. Science 345:1251788. 10.1126/science.1251788 25035500

[ref27] Isidro J. Jannink J. L. Akdemir D. Poland J. Heslot N. Sorrells M. E. (2015). Training set optimization under population structure in genomic selection. Theor. Appl. Genet. 128, 145–158. 10.1007/s00122-014-2418-4, PMID: 25367380 PMC4282691

[ref28] Jonas E. De Koning D. J. (2013). Does genomic selection have a future in plant breeding? Trends Biotechnol. 31, 497–504. 10.1016/j.tibtech.2013.06.003, PMID: 23870753

[ref29] Jordan K. W. Wang S. He F. Chao S. Lun Y. Paux E.. (2018). The genetic architecture of genome-wide recombination rate variation in allopolyploid wheat revealed by nested association mapping. Plant J. 95, 1039–1054. 10.1111/tpj.14009, PMID: 29952048 PMC6174997

[ref30] Koch P. Wujek B. Golovidov O. Gardner S. (2017). “Automated hyperparameter tuning for effective machine learning” in proceedings of the SAS Global Forum 2017 Conference. Carry, NC, 1–23.

[ref31] Lanning S. P. Talbert L. E. McGuire C. F. Bowman H. F. Carlson G. R. Jackson G. D. (1994). Registration of ‘McNeal’ wheat. Crop Sci. 34, 1126–1127. 10.2135/cropsci1994.0011183x003400040060x

[ref32] Lecun Y. Bengio Y. Hinton G. (2015). Deep learning. Nature 521, 436–444. 10.1038/nature14539, PMID: 26017442

[ref33] Li B. Zhang N. Wang Y. G. George A. W. Reverter A. Li Y. (2018). Genomic prediction of breeding values using a subset of SNPs identified by three machine learning methods. Front. Genet. 9:237. 10.3389/fgene.2018.00237, PMID: 30023001 PMC6039760

[ref34] Liu Y. Wang D. He F. Wang J. Joshi T. Xu D. (2019). Phenotype prediction and genome-wide association study using deep convolutional neural network of soybean. Front. Genet. 10:1091. 10.3389/fgene.2019.01091, PMID: 31824557 PMC6883005

[ref35] Lorenz A. J. Chao S. Asoro F. G. Heffner E. L. Hayashi T. Iwata H. (2011). “Genomic selection in plant breeding: knowledge and prospects” in Advances in agronomy. 1st Edn. Vol. 110 ed. Sparks D. L. (Cambridge: Academic Press), 77–123.

[ref36] Lorenzana R. E. Bernardo R. (2009). Accuracy of genotypic value predictions for marker-based selection in biparental plant populations. Theor. Appl. Genet. 120, 151–161. 10.1007/s00122-009-1166-3, PMID: 19841887

[ref37] Lozada D. N. Carter A. H. (2019). Accuracy of single and multi-trait genomic prediction models for grain yield in US Pacific northwest winter wheat. Crop Breed Genet. Genom. 1:e190012. 10.20900/cbgg20190012 PMC6981971 31881728

[ref38] Ma W. Qiu Z. Song J. Li J. Cheng Q. Zhai J.. (2018). A deep convolutional neural network approach for predicting phenotypes from genotypes. Planta 248, 1307–1318. 10.1007/s00425-018-2976-9, PMID: 30101399

[ref39] Maenhout S. De Baets B. Haesaert G. Van Bockstaele E. (2007). Support vector machine regression for the prediction of maize hybrid performance. Theor. Appl. Genet. 115, 1003–1013. 10.1007/s00122-007-0627-9, PMID: 17849095

[ref41] Mcdowell R. M. (2016). Genomic selection with deep neural networks. [graduate theses and dissertations], 50.

[ref42] McKay M. D. (1992). “Latin hypercube sampling as a tool in uncertainty analysis of computer models” in *Proceedings of the 24th Conference on Winter Simulation*; December 1992; 557–564.

[ref43] Meuwissen T. H. E. Hayes B. J. Goddard M. E. (2001). Prediction of total genetic value using genome-wide dense marker maps. Genetics 157, 1819–1829. PMID: 11290733 10.1093/genetics/157.4.1819 PMC1461589

[ref44] Min S. Lee B. Yoon S. (2017). Deep learning in bioinformatics. Brief. Bioinform. 18, 851–869. 10.1093/bib/bbw068, PMID: 27473064

[ref45] Montesinos-López O. A. Martín-Vallejo J. Crossa J. Gianola D. Hernández-Suárez C. M. Montesinos-López A.. (2019a). A benchmarking between deep learning, support vector machine and Bayesian threshold best linear unbiased prediction for predicting ordinal traits in plant breeding. G3 (Bethesda) 9, 601–618. 10.1534/g3.118.200998, PMID: 30593512 PMC6385991

[ref46] Montesinos-López O. A. Martín-Vallejo J. Crossa J. Gianola D. Hernández-Suárez C. M. Montesinos-López A.. (2019b). New deep learning genomic-based prediction model for multiple traits with binary, ordinal, and continuous phenotypes. G3 (Bethesda) 9, 1545–1556. 10.1534/g3.119.300585, PMID: 30858235 PMC6505163

[ref47] Montesinos-López O. A. Montesinos-López A. Crossa J. Gianola D. Hernández-Suárez C. M. Martín-Vallejo J. (2018b). Multi-trait, multi-environment deep learning modeling for genomic-enabled prediction of plant traits. G3 (Bethesda) 8, 3829–3840. 10.1534/g3.118.200728, PMID: 30291108 PMC6288830

[ref48] Montesinos-López A. Montesinos-López O. A. Gianola D. Crossa J. Hernández-Suárez C. M. (2018a). Multi-environment genomic prediction of plant traits using deep learners with dense architecture. G3 (Bethesda) 8, 3813–3828. 10.1534/g3.118.200740, PMID: 30291107 PMC6288841

[ref49] Okeke U. G. Akdemir D. Rabbi I. Kulakow P. Jannink J. L. (2017). Accuracies of univariate and multivariate genomic prediction models in African cassava. Genet. Sel. Evol. 49:88. 10.1186/s12711-017-0361-y, PMID: 29202685 PMC5715664

[ref50] Pedregosa F. Michel V. Grisel O. Blondel M. Prettenhofer P. Weiss R. (2011). Scikit-learn: machine learning in python. J. Mach. Learn. Res. 12, 2825–2830.

[ref51] Pérez P. de Los Campos G. (2014). Genome-wide regression and prediction with the BGLR statistical package. Genetics 198, 483–495. 10.1534/genetics.114.164442, PMID: 25009151 PMC4196607

[ref52] Pérez P. de los Campos G. Crossa J. Gianola D. (2010). Genomic-enabled prediction based on molecular markers and pedigree using the bayesian linear regression package in R. Plant Genome 3:106. 10.3835/plantgenome2010.04.0005, PMID: 21566722 PMC3091623

[ref53] Pérez-Enciso M. Zingaretti L. M. (2019). A guide for using deep learning for complex trait genomic prediction. Genes 10:553. 10.3390/genes10070553, PMID: 31330861 PMC6678200

[ref54] Pérez-Rodríguez P. Gianola D. González-Camacho J. M. Crossa J. Manès Y. Dreisigacker S. (2012). Comparison between linear and non-parametric regression models for genome-enabled prediction in wheat. G3 (Bethesda) 2, 1595–1605. 10.1534/g3.112.003665, PMID: 23275882 PMC3516481

[ref55] Pilgrim M. Willison S. (2009). Dive into python 3. Vol. 2 New York: Apress.

[ref56] Poland J. (2015). Breeding-assisted genomics. Curr. Opin. Plant Biol. 24, 119–124. 10.1016/j.pbi.2015.02.009, PMID: 25795171

[ref57] Poland J. Endelman J. Dawson J. Rutkoski J. Wu S. Manes Y. (2012). Genomic selection in wheat breeding using genotyping-by-sequencing. Plant Genome 5, 103–113. 10.3835/plantgenome2012.06.0006

[ref59] Ramcharan A. McCloskey P. Baranowski K. Mbilinyi N. Mrisho L. Ndalahwa M.. (2019). A mobile-based deep learning model for cassava disease diagnosis. Front. Plant Sci. 10:272. 10.3389/fpls.2019.00272, PMID: 30949185 PMC6436463

[ref60] Rangarajan A. K. Purushothaman R. Ramesh A. (2018). “Tomato crop disease classification using pre-trained deep learning algorithm” in Procedia Comput. Sci. 133, 1040–1047. 10.1016/j.procs.2018.07.070

[ref58] R Core Team (2017). A language and environment for statistical computing. Vienna, Austria: R Foundation for Statistical Computing.

[ref61] Rutkoski J. E. Heffner E. L. Sorrells M. E. (2011). Genomic selection for durable stem rust resistance in wheat. Euphytica 179, 161–173. 10.1007/s10681-010-0301-1

[ref62] Sallam A. H. Endelman J. B. Jannink J. L. Smith K. P. (2015). Assessing genomic selection prediction accuracy in a dynamic barley breeding population. Plant Genome 8:eplantgenome2014.05.0020. 10.3835/plantgenome2014.05.0020, PMID: 33228279

[ref63] Samuel A. L. (2000). Some studies in machine learning. IBM J. Res. Dev. 44, 206–226. 10.1147/rd.441.0206

[ref74] Srivastava N. Hinton G. Krizhevsky A. Sutskever I. Salakhutdinov R. (2014). Dropout: a simple way to prevent neural networks from overfitting. J. Mach. Learn. Res. 15, 1929–1958. PMID: 25490985

[ref64] Sukumaran S. Dreisigacker S. Lopes M. Chavez P. Reynolds M. P. (2015). Genome-wide association study for grain yield and related traits in an elite spring wheat population grown in temperate irrigated environments. Theor. Appl. Genet. 128, 353–363. 10.1007/s00122-014-2435-3, PMID: 25490985

[ref65] Sun J. Poland J. A. Mondal S. Crossa J. Juliana P. Singh R. P.. (2019). High-throughput phenotyping platforms enhance genomic selection for wheat grain yield across populations and cycles in early stage. Theor. Appl. Genet. 132, 1705–1720. 10.1007/s00122-019-03309-0, PMID: 30778634

[ref66] Tishbirani R. (1996). Regression shrinkage and selection *via* the Lasso. J. R. Stat. Soc. Series B Stat. Methodol. 58, 267–288. 10.1111/j.2517-6161.1996.tb02080.x

[ref67] Wang H. Cimen E. Singh N. Buckler E. (2020). Deep learning for plant genomics and crop improvement. Curr. Opin. Plant Biol. 54, 34–41. 10.1016/j.pbi.2019.12.010, PMID: 31986354

[ref68] Wang S. Wong D. Forrest K. Allen A. Chao S. Huang B. E.. (2014). Characterization of polyploid wheat genomic diversity using a high-density 90 000 single nucleotide polymorphism array. Plant Biotechnol. J. 12, 787–796. 10.1111/pbi.12183, PMID: 24646323 PMC4265271

[ref69] Wang J. Zhou Z. Zhang Z. Li H. Liu D. Zhang Q.. (2018). Expanding the BLUP alphabet for genomic prediction adaptable to the genetic architectures of complex traits. Heredity 121, 648–662. 10.1038/s41437-018-0075-0, PMID: 29765161 PMC6221880

[ref70] Young S. R. Rose D. C. Karnowski T. P. Lim S. H. Patton R. M. (2015). “Optimizing deep learning hyper-parameters through an evolutionary algorithm” in Proceedings of the Workshop on Machine Learning in High-Performance Computing Environments; November 2015; 1–5.

[ref71] Zingaretti L. M. Gezan S. A. Ferrão L. F. V. Osorio L. F. Monfort A. Muñoz P. R.. (2020). Exploring deep learning for complex trait genomic prediction in polyploid outcrossing species. Front. Plant Sci. 11:25. 10.3389/fpls.2020.00025, PMID: 32117371 PMC7015897

[ref72] Zou H. Hastie T. (2005). Addendum: regularization and variable slection *via* the elastic net. J. R. Stat. Soc. Series B Stat. Methodol. 67:768. 10.1111/j.1467-9868.2005.00527.x

[ref73] Zou J. Huss M. Abid A. Mohammadi P. Torkamani A. Telenti A. (2019). A primer on deep learning in genomics. Nat. Genet. 51, 12–18. 10.1038/s41588-018-0295-5, PMID: 30478442 PMC11180539

