# Supplementary material for: Deep Learning for Predicting Complex Traits in Spring Wheat Breeding Program
Source: Front Plant Sci. 2021 Jan 5;11:613325. doi: 10.3389/fpls.2020.613325 (PMC7813801; doi:10.3389/fpls.2020.613325)
Supplement: Supplementary file 1 [file Table_1.docx]

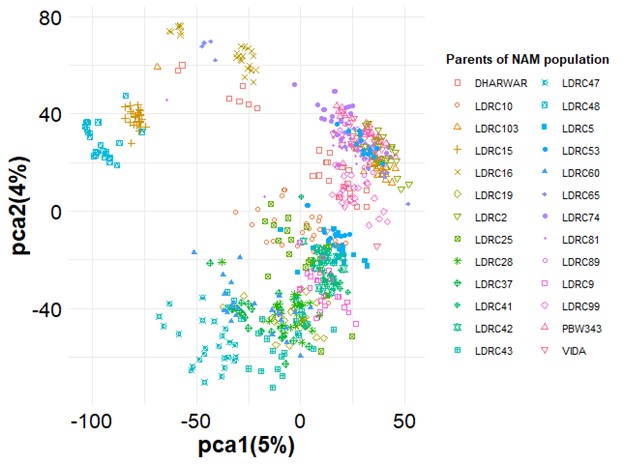


**Supplementary Figure 1**. Principal component analysis of the NAM population containing 26 diverse founder parents. The first PC explains 5% and second PC explains the 4% of the total variation present in the population.


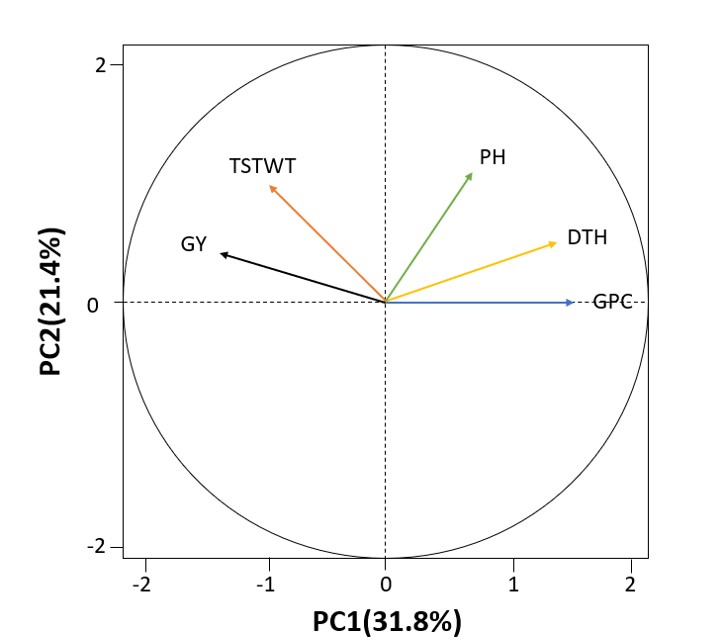


**Supplementary Figure 2.** Principal component analysis of the five phenotypic traits evaluated in this study. The first and second PC explains the 31.8% and 21.4% of the total phenotypic variation observed. This figure further confirms the presence of negative correlation between the grain yield and grain protein content.

| **Supplementary Table 1.** Average values of agronomic traits evaluated in this study for three environments (2014-2016) at Spillman Agronomy Farm, Pullman, WA. | | | | | |
| --- | --- | --- | --- | --- | --- |
| Environment | Grain yield (t/ha) | Grain protein content (%) | Test weight (lb) | Plant height (inches) | Days to heading (Julian days) |
| 2014 | 2.0 | 14.4 | 58.0 | 38.2 | 179.0 |
| 2015 | 1.7 | 12.2 | 59.3 | 35.8 | 163.1 |
| 2016 | 2.4 | 12.6 | 61.6 | 35.2 | 169.3 |
